# "It's sort of like being a detective": Understanding how Australian men self-monitor their health prior to seeking help

**DOI:** 10.1186/1472-6963-8-56

**Published:** 2008-03-14

**Authors:** James A Smith, Annette Braunack-Mayer, Gary Wittert, Megan Warin

**Affiliations:** 1 Discipline of Public Health, School of Population Health & Clinical Practice, University of Adelaide, Level 9 – Tower Building (MDP 207), 10 Pulteney St, Adelaide SA 5005, Australia; 2 Discipline of Medicine, School of Medicine, University of Adelaide, Level 6 – Eleanor Harrald Building, Frome Road, Royal Adelaide Hospital, Adelaide SA 5005, Australia; 3 Department of Anthropology, University of Durham, 43 Old Elvet, Durham DH1 3HN, UK

## Abstract

**Background:**

It is commonly held that men delay help seeking because they are ignorant about and disinterested in their health. However, this discussion has not been informed by men's lay perspectives, which have remained almost entirely absent from scholarship relating to men's help seeking practices.

**Methods:**

In this qualitative paper, we draw on semi-structured interviews with 36 South Australian men to examine their understandings of help seeking and health service use.

**Results & Discussion:**

We use participants' talk about self-monitoring to challenge the assumption that men are disinterested in their health, arguing instead that the men in our study monitored their health status and made conscious decisions about when and how to seek help. Using an inductive approach during the thematic analysis we were able to identify four key factors that influenced how men monitored their health and explain how these intersect with the way men sought help and used health services.

**Conclusion:**

We show that the men in our study were actively engaged in the self-monitoring of their health. We suggest that these findings offer an alternative approach for understanding how we can promote men's interaction with health services.

## Background

Men's reluctance to seek help and use health services is a concern across most Western cultures [1-4]. Some commentary has suggested that men are victims of their own behaviour [5-9]. This has been used to argue that men are ignorant about or disinterested in their health [10-12]. These conceptions have often been linked to hegemonic masculine traits that place an expectation on men to be independent, strong, stoical and tough [7,13-15].

Building upon recent literature which has challenged this victim-blaming mentality [4,16-22], this paper draws on qualitative research to argue that men are actively engaged in monitoring their health prior to seeking help. Following a discussion of the study context, the first section examines what men mean by self-monitoring their health through a discussion of how and why men seek different types of information, from different sources, to monitor their health. We then outline the factors that influence men's help seeking practices. These factors, which include the length of time available to monitor health and legitimate help seeking; previous illness experiences; maintaining regular activities; and an assessment of illness severity, point to the ways in which men rationalize and enter into help seeking.

## Methods

### Study context

Our research forms part of the Florey Adelaide Male Ageing Study (FAMAS) at the University of Adelaide [23]. The cohort consists of 1,195 participants aged between 35 to 80 years of age randomly drawn from the North-West Adelaide region in South Australia using the white pages telephone directory [23]. From the larger sample, and using strata relating to age and marital status, we invited 36 men to participate in a qualitative study exploring men's help seeking behaviors and health service use. Additional demographic information relating to this smaller sample can be found in Smith et al [24].

### Conducting interviews

The study was approved by the University of Adelaide Human Research Ethics Committee, and informed consent was obtained from each participant. Interviews lasted between one and one and three quarter hours. The first author conducted all in-depth interviews. We carried out our interviews away from traditionally feminised environments in an effort to preserve our participant's masculine identities [25,26]. This included avoiding health services where men were likely to feel threatened or alienated [27], resulting in the majority of interviews being conducted at the homes of our participants. There were four occasions where a university interview room or a participant's workplace were used as a venue, at the request of participants.

We used a semi-structured interview format to encourage open-ended discussion among our participants. We found that this provided a flexible framework for interviews to be guided by the discussion of participants. We began most interviews by asking participants a little bit about themselves, often eliciting responses relating to family and work. This was useful for developing rapport with participants, and provided scope for further questioning about how family and work relationships influenced their health. We then proceeded to ask participants' about what they perceived was most important to them about their health. This provided a meaningful context for a more detailed exploration of their help seeking practices and health service use.

We found that the men in our study were willing to speak about their health in an open manner when provided with an appropriate environment in which to do so. Rapport building was central to achieving this outcome. Rapport was built by sharing mutual experiences and showing an interest in topics that appear to be unrelated to the research intent such as hobbies, leisure pursuits and relationships with friends and family. Rapport building also extended to sharing a beer with participants, watching family videos and sharing stories of stereotypical masculine endeavours such as engaging in risk-taking behaviours. Engaging our participants in this way helped them to be articulate about a range of health concerns, including those that are traditionally considered to be stigmatising among most groups of men. However, it should also be noted that building rapport within the interview context by adopting traditionally masculinised behaviours – such as drinking beer and talking about risk taking – may implicitly reinforce negative health practices among men, akin to similar critiques in contemporary men's health promotion work [16,22,28]. Likewise, the perpetuation of stereotypical masculine endeavours during research interviews does little to affect the 'patriarchal dividend' that advantages men through the subordination of women and/or marginalised groups of men, hence may actually reinforce structural gendered inequalities (albeit an unintentional consequence) [28]. Nevertheless, we recognise that there are both strengths and weaknesses associated with building rapport in this way and that further reflexive accounts examining the intersection between men, masculinity and research interviews, are required, but extend beyond the scope of this paper (see for example Robertson 2006) [28].

Each interview was transcribed verbatim, with field notes included where necessary. The first author coded the interview data using NVIVO software, using an inductive approach during the thematic analysis.

## Results & Discussion

### What does self-monitoring mean to men?

This section describes what the men in our study mean by self-monitoring their health. Our findings suggest that self-monitoring is a health practice informed by the health information men gather when responding to health problems. We examine the way in which our participants seek different types of information (including 'scientific' knowledge), from different sources, to enable them to make an informed decision as to whether they should seek help.

There is a range of terms that can reflect how men monitor their health. These can include 'self-assessment', 'self-surveillance' and 'self-monitoring'. In this paper we have opted to use the term 'self-monitoring', for two reasons. Firstly, this prevents confusion with nomenclature in other disciplines: the way in which 'self-assessment' is described in epidemiological work [29,30]; how 'surveillance' is described in sociological scholarship, particularly that with a theoretical orientation [31]; and how 'self-surveillance' has emerged as a hybrid term between epidemiology and sociology [32]. Secondly, 'self-monitoring' is a term which we consider seems most closely to reflect the way in which our participants contextualised and spoke about their help seeking practices. We now examine what self-monitoring meant to the men in our study.

Self-monitoring, as defined by our participants, is a health practice that often precedes help seeking. For example, Alexander commented:

You've got to be aware that you've got a problem. Obviously that's the first thing. And then you've got to assess as to whether or not you can fix it yourself, or find somebody who knows more about it than you do. (Alexander, 75)

For Alexander, self-monitoring was highly dependent, in the first instance, upon recognising that a health problem exists. This was reinforced by Robert, who said:

I know my stupid body by now. I know if something strange happens to me, but I also need to know what's basically going on, to fix it myself. (Robert, 59)

The nexus between knowing one's body, recognising that a health problem exists and being able to 'fix' a health problem is complex. The majority of the men in our study approached help seeking in a conscious manner – it was not just something they did, but something they thought about. Our participants consistently discussed the need to make an informed decision when opting to seek help. Previous literature has suggested that informed choices are based on relevant knowledge, consistent with the decision maker's values, and can be behaviourally implemented [33]. To make an informed decision our participants asked questions and gathered information about their health concerns. For example, when Charlie sought information for a degenerative muscle condition, he stated:

At the end of the day I think you've got to find out for yourself. And the only way you find out for yourself is to ask questions. I've never been afraid to go and ask questions...I'm the sort of person who likes to do my research. I like to make sure that I've sought other people's opinions where necessary... I've sought opinions from doctors, and I've read quite a lot of medical books on musculoskeletal dysfunction. And I refer to them quite a lot...this helps me gauge my own health. (Charlie, 54)

Charlie gathered information in various ways. He questioned others, such as health service providers and partners – hence information gathering can be a relational activity. He also read books and conducted internet searches – hence information gathering can also be an individual pursuit. However, the ultimate *decision *to seek help lies with the *individual*. Having the capacity to gather and order health information to make an informed decision was important to the men in our study. Rhys described this process by commenting:

I sort of look at a problem, look at what's really going on...to get the facts, to marshal the facts, to consider what the best options are...and to take responsibility to make decisions...full responsibility for my health, not partial, full. Because you know, I make the decision to do it [use health services]. (Rhys, 57)

Rhys' comments highlight the fact that self-monitoring for the men in our study involved the acquisition of information that they perceived to be factual. They then used this information to decide whether to seek help. As Tim commented:

I sort of like to know what's going on. Like scientifically. I quite like science, even if I don't understand it...I like to analyse. It's sort of like being a detective...you're trying to work out what's going on. (Tim, 52)

Both the collation of facts, and the analysis of what constitutes those facts, contribute to what self-monitoring meant to the men in our study. As Tim suggested, self-monitoring is like being a detective – an endeavour to understand 'what's going on'.

### From self-monitoring to help seeking

Our participants gathered information for a range of purposes. We have already demonstrated that self-monitoring is a health practice central to the way the men in our study interpreted their need to use health services. We now extend this discussion to elaborate on the nexus between self-monitoring and help seeking for these men. We draw on a prominent 'fix-it' discourse to demonstrate that the main objective of our participants was to return to a familiar state of health to live a 'normal' life. We identify four key factors of self-monitoring that influence men's help seeking practices, and show how these are perceived to contribute to fixing the health concerns of our participants. These factors relate to (i) the length of time available to monitor health and legitimate help seeking; (ii) men's previous illness experiences; (iii) how men monitor their health in relation to their ability to maintain regular activities in the context of their daily lives – such as being able to pursue leisure activities, maintain work roles, and complete functional tasks such as driving the car; (iv) the way men monitor their health status with respect to illness severity – incorporating issues such as the time of onset of ill health, the type of health concern being monitored and the presence of pain.

When asked "what has made you seek help in the past?" Edward (aged 46) casually stated "something I couldn't fix myself". Like most participants, Edward's motivation to seek help related to being able to fix a health concern. This was also demonstrated by Tim, who suggested:

Anything that persists, that I can't fix myself, I'll definitely go and see the doctor...I'd put up with it for about a week initially, and then that's about it. Then I'll think 'ok, something is wrong here'. (Tim, 52)

In the first instance, Tim spoke about trying to fix a health concern himself. He then explained how he would seek professional help for the health concern if he perceived that he was unable to fix it himself. Tim's comments show that identifying a health problem, engaging in self-monitoring and deciding whether or not to seek help is a multifaceted process. It depends on a range of factors, one of which is the length of time available to make a choice about health maintenance.

#### Length of time available to monitor health and legitimate help seeking

Time consistently emerged as an important factor when the men in our study were deciding whether they could fix the problem themselves or whether they needed to seek professional help. The time men took to monitor their health often depended on the type of health concern being assessed and whether (or not) they perceived that seeking help would improve the final health outcome. As Steve stated:

I prefer my body to um, you know, have a chance to rest and fix itself. Going to your local GP, they don't necessarily have a wand that they can wave over you to fix you. (Steve, 38)

Steve justified waiting to seek help in two ways. Firstly, he suggested that in order to self-monitor his health he needed to let his body rest. In this instance, rest equated to allowing his body to fix itself. Secondly, he implied that visiting a doctor prematurely did not necessarily result in his health problems being fixed. Steve could justify a prolonged self-monitoring period indicating that he was not disinterested in his health. Rather, he had an acute awareness of and desire to self-monitor his health.

Steve's example highlights a potential contradiction between men wanting sufficient time to self-monitor their own health, and the expectation they have for health service providers to make an instant diagnosis to fix their health concerns when they do decide to seek help. This was also illustrated by Ron who stated:

I'd like a health service that operates like a car service – take it in, fix the problem, go home! (Ron, 57)

The immediacy required of health professionals to fix men's health problems is striking. Yet, time in the context of both self-monitoring and help seeking often intersected with other factors, such as the type of health concern being assessed. For example, when asked how concerned he would have to be before going to the doctor, Brad replied:

I guess it depends on the nature of the problem. Um, if it's a sore joint – and right now I have a sore elbow which has been bugging me for a while – I'll monitor it...because those sort of things, in my experience, sort themselves out ...But if it's getting worse, or nothing's changed, then I would say, 'well I've got to do something else'. But for that period I'm not just ignoring it. I'm sort of paying attention to it and working out whether or not it will sort itself out. (Brad, 38)

Brad's sore elbow was not something that he dismissed. Rather it was a concern that required ongoing monitoring – again, to see if it 'sorts itself out'. He used past experience to assess whether help seeking was required, in this case using it to delay help seeking, even if it ended up compromising his health status. This was mirrored by Paul who claimed:

They're [men] happy to wait for a period of time. Even if it means that it might increase their morbidity, which generally it does. (Paul, 40)

Men such as Paul recognised that delayed help seeking could result in poorer health outcomes. This does not, however, mean that the men in our study were disinterested in their health. In fact, most of our participants who expressed a reluctance to use health services were able to articulate clearly why they had chosen not to seek help. For example, when Harry was asked to describe how he sought help, he stated:

I feel relaxed to know that the [health] system is there if I did need help. I certainly wouldn't take a chance if I thought I had a serious problem. But I just don't consider that I should take advantage of the system. In other words, I wouldn't run down to the doctors if I've got a headache or something like that. To me you're literally wasting his [doctor's] time and your own...unless of course it was a headache that dragged on for three or four days or something like that. Something you couldn't fix.

(Harry, 68)

While Harry acknowledged that he might delay help seeking for some health concerns, such as a headache, he also explained why – noting the seriousness of the health concern and the length of time over which the health problem manifested. Interestingly, the same reasons were used by other men to legitimize their decisions to seek help. For example, Clancy commented:

I tend to think if you have an accident, and break your leg, that you're going to get it fixed, and looked after, straight away...I would like to think that if something was wrong, then yes, they're [doctors] there to fix you up and give you the best treatment as quickly as possible. (Clancy, 53)

Health concerns such as a broken leg were perceived to require immediate attention. Chest pain was another example that was occasionally mentioned as requiring instant attention. For example, Trent stated:

I went into the hospital. They took me in straight away and checked me out. They keep you there for about two hours. Then, 'oh no, you're ok, you can go home' and I said 'I feel silly coming over here [hospital] for nothing', They said 'don't ever feel like you're silly, because if we can get you in here and you're not right, we can fix you up straight away'. So now it doesn't worry me, if I'm not feeling right I head straight down and see them [local hospital emergency department]. (Trent, 69)

Previous research has found that men delay seeking help while attempting to rationalize the symptoms of chest pain [34]. However, our study findings suggest that, if health service providers can influence that period of rationalization, such as by providing positive reinforcement during previous health encounters, then men may well seek help for their chest pain. The validation provided by Trent's health service providers at his local hospital emergency department resulted in him making future decisions to seek help more readily. This confirms that it is not only the self-monitoring period that influences the help seeking decisions that men make, but the reactions of health service providers during previous health encounters as well.

#### Previous illness experiences

Previous bouts of ill health and injury were frequently referred to when monitoring a health concern. This was particularly true in instances where the severity of a health concern was being assessed. For example, Percy commented:

I tend to get around home with my thongs on a lot. And a couple of times, you know, you go to walk from one room to the other and you short change the corner, and oh!!! You bend your little toe back and it's sore, it swells up and everything. And you think, oh, I've broken that. Perhaps I should go to the doctor. Then it comes good after a week, you know. So why bother going? (Percy, 60)

Recognising that his toe usually 'comes good after a week' made Percy decide not to seek professional help. The men in our study adopted this 'commonsense approach' when monitoring their health. This approach varied with the type of health concern being monitored. As Rhys commented:

The last time I was ill I had some spates of diarrhea – probably three. The last one was a week ago. I got that on a Monday after drinking a bottle of my son's home brew actually. And by Friday it hadn't cleared up so I went to the doctor. Coz I'd never had diarrhea for that long before. And then the doctor's advice was 'well, if it's still there in ten days after the onset perhaps we should look further'. But eventually it went away. (Rhys, 57)

Rhys waited four days before seeking help; it was the unexpected and prolonged duration of symptoms that caused him concern. Again, illness severity was assessed through previous experience. In Rhys' case, the abnormality of his bodily functions led him to seek help from his doctor. Similarly, Robert stated:

Now I know when the gout is coming back. I've had it for so many years I know when it's coming on in my ankles. I can control it myself. (Robert, 59)

Robert's ability to comprehend what was happening to his body was based on previous experiences. These previous experiences subsequently legitimated his decision not to seek professional help. In this case, it was something he perceived that he could control himself. Other participants perceived the inability to control bodily functions themselves as reason for seeking help, particularly if managing a chronic condition such as diabetes. As Conrad commented:

I guess being diabetic I tend to watch it (being ill) more closely. And I'll do something about it more quickly than I normally would, coz it may affect my blood sugar...especially if I find my blood sugar is going up. Like if I've got a cold and the blood sugar remains pretty much the same as it has, then I'm quite happy not to go (to the doctor)...as soon as I see that my blood sugar is going up I go to the local doctor and get him to check it out. If you've got an infection, then don't leave it too long coz it might just get worse. It's better to get it under control (Conrad, 63)

In this instance, Conrad used his blood sugar measurements to assess his diabetes. In turn, he used this measurement to assess other aspects of his health, and used this as a basis for deciding whether to seek help. Charlie also recognised that he monitored his health more rigorously after he had been diagnosed with diabetes, by suggesting:

I've gone back to doing what I consider to be more consistent checks. Because they told me the dangers of the things that can happen through your diabetes, and that you can get off track really quickly. So unless you monitor it, you don't know where you're going. (Charlie, 54)

Both Conrad & Charlie's excerpts indicate that the presence of a chronic condition resulted in more detailed monitoring of their health, at least in comparison to what had occurred in periods prior to developing their chronic illness. As Charlie mentioned, it was about 'staying on track'.

#### Maintaining regular activities

The ability to maintain regular activities was important to our participants. They frequently used this to monitor their health. This is consistent with Robertson's work relating to embodied masculinity which highlights that health practices – such as help seeking – are rarely central to men's lives but are usually dependent upon, and secondary to, other aspects of their daily life [19,21,35]. Inevitably this involves men conceptualizing their health and illness in relation to their ability to carry out daily tasks or maintain regular activities. When viewed in relation to men's health practices, such as help seeking, this process is referred to as pragmatic embodiment and can be defined as the functional use of a 'normal everyday body' to fulfill specific social roles – such as father, husband and worker [19,21].

The nature of these regular activities differed markedly among the men in our study – dependent upon age, marital status, employment history, and many other factors. This is consistent with recent men's health scholarship indicating that there are serious limitations associated with perceiving men as a homogenous population [20,22]. For example, Sam said:

Going to the chiropractor first is not necessary...if I move I get a twinge (lower back) every now and again. The boat will test it out tomorrow anyway. Yeah, the boat will test it out tomorrow. (Sam, 74)

Sam went on to explain how during his retirement he enjoyed being able to go out on regular fishing trips. For him, being able to fish, including being able to launch his fishing boat, was a primary part of leading a normal life. His back pain was secondary to being able to lead that life, but a concern nonetheless. Sam used his ability to launch his fishing boat to monitor whether he needed to make an appointment to see his chiropractor.

Similar scenarios emerged throughout our study. For instance, when Brad was questioned about the last time he was sick, he said:

In terms of being ill (pause), probably last time I was really, really sick, and I guess by definition of sick, that is when I can't actually go scuba diving. That probably would have been about five, maybe four years ago. (Brad, 38)

Understanding that Brad is a casual scuba diving instructor explains why he assessed his health in this way. He went on to describe how the inability to maintain this regular activity affected his capacity to earn an income (work role), which in turn limited his capacity to financially support his daughter (fathering role).

The ability to maintain regular activities also influenced our participants' capacity to seek help and access health services. For example, Clancy commented:

When I have migraines, I get blotchy eyes. I get stars and I can't see. So basically I can't do anything. I can't walk. I can't drive. All that happens within half an hour. So as soon as it starts I've got to get somewhere very, very quickly, or I've got to stay at home, or go to the medical centre at work. (Clancy, 53)

Clancy could recognise the symptoms he develops when he experiences a migraine. He went on to explain how the inability to walk and drive limited his capacity to seek help and he also described strategies that he uses to overcome his migraines. Likewise, when speaking about driving a car to his local health service for severe stomach pain, Andrew commented:

I can still drive the car down there...I've never been bad enough for my wife to have to take me – probably once in about 10 years. (Andrew, 47)

When the men in our study did not seek help, it was usually because they were unable to do so. They were also aware of the burden they placed on significant others, such as their wives, if they decided to seek help. That is, the practice of help seeking – particularly that relating to seeking a professional opinion – was a relational task. In fact, men's social contexts and relationships have a direct bearing on how they self-monitor their health and how they decide to seek help [36]. If health service providers and significant others recognise that men assess their health against being able to maintain regular duties, then discussion prior to and during health encounters, and the solutions offered to 'fix' men's health concerns, can be directed towards the way men conceptualise their health at an individual level.

Using regular activities as a yardstick does not necessarily improve health outcomes for men or their ability to maintain their health, and/or recover from illness. For example, George mentioned:

About 20 years ago I had a real bad dose of the flu. I had it for 1 month. I couldn't get out of bed, except to feed my cat at the time. I was completely bed ridden. I couldn't even get up to go and see a doctor. I would have loved the doctor to have come to me. I just rested and stayed in bed. I think I went back to gardening too early and it aggravated it a little, it prevented a good recovery. (George, 64)

George used bed rest to overcome his bout of flu, yet he also recognised that his desire to do some gardening interfered with his recovery. Some men in our study, like George, were able to recognise that the decisions they made did not improve their health. This highlights how the men in our study balanced their health and health status against other aspects of their lives. Where men do place other aspects of their lives ahead of their health, we need to better understand why.

#### Perceived illness severity

Illness severity was a key factor influencing the way that men monitored their health, and subsequently sought help and used health services. Illness severity was measured in a variety of ways. It could relate to the time of onset of the illness or health concern, and whether the health issue persisted over a prolonged period. It could relate to the type of health concern being monitored – ranging from a stubbed toe to diarrhea, or diabetes to chest pain (as already discussed). It could also be measured through pain or visible physical impairments such as cuts or bruising. If an illness or injury was perceived to be visually disturbing or life threatening from its onset, then professional help was generally sought sooner rather than later. For example, Clancy reflected back on an injury that he had sustained when renovating his house by stating:

I was taking my trailer down the driveway...and the trailer got away from me, hit the wall, and fell into the trailer. I cut all my face, had black eyes and all that. I went to the doctors and got myself checked out. So when you tend to know that you're hurt, you go. I've chased up things like that...when it's more visible. You see it and sort of do something about it. (Clancy, 53)

As Clancy indicated above, the physicality and visibility of health concerns such as cuts and bruising resulted in an action-oriented judgment relating to help seeking, and a shorter than usual monitoring period. Health concerns involving pain were accorded a similar level of immediacy. Percy aged 60, stated "I really only go when I get a pain or an ache somewhere".

The intersection between time and pain was important for how the men in our study monitored their health. The time needed to monitor pain differed markedly between participants depending on the health concern being assessed. Persistent or on-going pain was perceived to be a significant marker of illness severity, and one that often required the help of a health professional. For example, when commenting on a tooth-ache, Gareth mentioned:

The wisdom teeth were up. They'd been up for ages. But the next thing I know was that the gum was growing over one of the teeth, and it became inflamed and every time I went to bite it became even more inflamed. And it was getting bigger, and it went in a vicious circle it just got bigger and bigger, and more and more tender. So eventually I thought hell, I'm going to have to see a dentist. (Gareth, 40)

Charlie reiterated this concept by reflecting on his neck pain:

I had to go and have surgery, a spinal fusion on my neck. I took myself up there as an in-patient, but I had that much pain in my arm I thought something had to have gone wrong. So I admitted myself to hospital. (Charlie, 54)

Likewise, Harry mentioned that severe stomach pain would also necessitate seeking help from a health professional, by stating:

If I thought that I had a problem that I should seek medical advice for, I wouldn't hesitate. I wouldn't hesitate. If I was getting a very bad pain in my stomach or something like that, and what have you, I'd say well if you don't get this sorted out you could be in trouble. (Harry, 68)

These men were inclined to seek professional help for a range of health concerns if there was evidence of pain. This contrasts stereotypical conceptions of hegemonic masculinity outlined in the introduction of this paper [5,9-11,37]. In fact, there were only a small minority of men in our study whose health practices reflected hegemonic masculinity. For example, when discussing severe back pain Claude said:

When the back goes out, I mean 'oh Jesus', you can't sit, you can't stand, you can't walk. I mean I've laid in bed for five weeks. If I want to go to the toilet I crawl to the toilet, and if I want to get something to eat I crawl down here [kitchen] and get something to eat. I don't expect anyone to drop everything, and I mean I can cope with it, I know when I can't cope with it, then I'll ask for help. (Claude, 59)

While Claude clearly wanted to maintain his independence by not having to rely on others, he ultimately acknowledged that if he "can't cope with it" then he'll "ask for help". He recognized that he was not infallible and stated that there would be a point in time when he would need to seek help. It was not merely a matter of soldiering on. Rather it was a matter of balancing the various factors that shape his understanding of when he should or should not seek help.

## Conclusion

Drawing on men's lay perspectives of their help seeking practices, we have described how 36 men residing in North-Western Adelaide region of South Australia self-monitored their health prior to seeking professional help. We have argued that our research presents a different picture from previous research which has focused, almost exclusively, on men's apparent reluctance to seek help out of indifference. By contrast, our participants did self-monitor their health and illness, and this influenced the decisions they made to seek professional help for their health concerns. Our findings suggest a different framework is needed to understand help seeking practices and health service use among the men in our study.

Periods of self-monitoring can point toward a high degree of interest in, and reflective thought about, one's health. Health service providers and policy-makers need to take men's self-monitoring behaviour into account when attempting to engage men within the health system [21,35,38,39]. In the context of this paper, this means responding to the way in which individuals or sub-populations of men self-monitor their health. This also requires health service providers and key decision-makers to reflect critically on the ways in which they have positioned men, and their respective health practices, in the past. We should not assume that current health services meet the needs of men, or that health service providers or policy-makers are appropriately geared to improve men's use of health services [4]. Our conclusions are consistent with recent international scholarship which has called for health professionals and policy makers to pay much closer attention to men's health and illness experiences, particularly those that relate to the way in which men learn from and listen to their bodies [19,21,39].

Recognising that men attempt to gather credible health information in order to self-monitor health concerns is an important concept for health service providers to consider. The men in our study preferred to access health information prior to seeking help, and this provides an opportunity to build the critical health literacy of men [40,41]. This has the potential to empower men to make informed decisions about their health and to take responsibility for health-related decisions. It also places an expectation on the public health system to provide and disseminate health information in a format that is accessible and meaningful to men and to provide health services that are respectful of, and that respond to, the social circumstances influencing the health of men.

Our analysis has identified four factors that shape self-monitoring and help-seeking among men: length of time available to monitor health and legitimate help seeking; previous illness experiences; capacity to maintain regular activities and every day tasks; and perception of the severity of health concerns. We are not suggesting this is a definitive list of factors to be considered in developing meaningful strategies to engage men with health services. Nor are we suggesting that the only health practice important to men is self-monitoring their health, as clearly it is not. However, we are suggesting that paying closer attention to the ways men approach their health challenges traditional stereotypes associated with health practices among men, particularly those that deviate from a hegemonic view of help-seeking [3]. Listening to men's lay perspectives can contribute to policy and practice responses relating to men's health [21,35,39]. We acknowledge, however, that there are limitations to the generalisability of exploratory research of this nature. Men's health practices will differ according to social, cultural and environmental contexts. As such, we encourage men's health researchers in other settings to pursue additional work in this area.

To conclude, the men in our study who self-monitored their health were aware of, and had a genuine interest in, their health and wellbeing. This re-affirmed for us that it is unproductive to perpetuate and maintain a victim blaming approach in men's health research. Re-conceptualising how men negotiate their health and health practices, by listening to their lay perspectives, is a crucial component for advancing men's health research, practice and policy responses at local and global levels. This, in turn, has the potential to develop health systems that are capable of engaging men in more meaningful discussion about their health. This will ultimately contribute to enhancing the quality and longevity of men lives.

## Competing interests

The author(s) declare that they have no competing interests.

## Authors' contributions

JS reviewed relevant literature, conducted all interviews, coded and analysed the data, and completed the first draft of the paper. ABM provided supervision during the coding and interpretation of the data, and assisted with the drafting and refinement of the paper. GW is the Chief Investigator of FAMAS, provided supervision during all aspects of the study and critically revised the content of the paper. MW is a Principal Investigator of FAMAS, supervised the co-ordination of the data collection (fieldwork), contributed to the design of the semi-structured interview schedule and critically revised the content of the paper. All authors read and approved the final manuscript.

## Pre-publication history

The pre-publication history for this paper can be accessed here:


